# Predicting AD Conversion: Comparison between Prodromal AD Guidelines and Computer Assisted PredictAD Tool

**DOI:** 10.1371/journal.pone.0055246

**Published:** 2013-02-12

**Authors:** Yawu Liu, Jussi Mattila, Miguel Ángel Muñoz Ruiz, Teemu Paajanen, Juha Koikkalainen, Mark van Gils, Sanna-Kaisa Herukka, Gunhild Waldemar, Jyrki Lötjönen, Hilkka Soininen, for The Alzheimer’s Disease Neuroimaging Initiative

**Affiliations:** 1 Department of Neurology, University of Eastern Finland, Kuopio University Hospital, Kuopio, Finland; 2 Department of Clinical Radiology, University of Eastern Finland, Kuopio University Hospital, Kuopio, Finland; 3 VTT Technical Research Centre of Finland, Tampere, Finland; 4 Department of Neurology, Memory Disorders Research Group, Rigshospitalet, Copenhagen University Hospital, Copenhagen, Denmark; Banner Alzheimer’s Institute, United States of America

## Abstract

**Purpose:**

To compare the accuracies of predicting AD conversion by using a decision support system (PredictAD tool) and current research criteria of prodromal AD as identified by combinations of episodic memory impairment of hippocampal type and visual assessment of medial temporal lobe atrophy (MTA) on MRI and CSF biomarkers.

**Methods:**

Altogether 391 MCI cases (158 AD converters) were selected from the ADNI cohort. All the cases had baseline cognitive tests, MRI and/or CSF levels of Aβ1–42 and Tau. Using baseline data, the status of MCI patients (AD or MCI) three years later was predicted using current diagnostic research guidelines and the PredictAD software tool designed for supporting clinical diagnostics. The data used were 1) clinical criteria for episodic memory loss of the hippocampal type, 2) visual MTA, 3) positive CSF markers, 4) their combinations, and 5) when the PredictAD tool was applied, automatically computed MRI measures were used instead of the visual MTA results. The accuracies of diagnosis were evaluated with the diagnosis made 3 years later.

**Results:**

The PredictAD tool achieved the overall accuracy of 72% (sensitivity 73%, specificity 71%) in predicting the AD diagnosis. The corresponding number for a clinician’s prediction with the assistance of the PredictAD tool was 71% (sensitivity 75%, specificity 68%). Diagnosis with the PredictAD tool was significantly better than diagnosis by biomarkers alone or the combinations of clinical diagnosis of hippocampal pattern for the memory loss and biomarkers (p≤0.037).

**Conclusion:**

With the assistance of PredictAD tool, the clinician can predict AD conversion more accurately than the current diagnostic criteria.

## Introduction

Alzheimer’s disease (AD) is the most common form of dementia in the elderly [1]. The pathology of AD starts years, even decades before any appearance of symptoms. The current hypothesis is that interventions should be started at an early phase in order to be efficient. Therefore, early diagnostics is essential 1) for detecting persons in clinical trials where pharmaceutical or psychosocial interventions are developed, and 2) for starting treatments at the earliest phase possible when efficient treatments become available in future. If one could have an intervention to delay disease onset or progression this would dramatically reduce the global burden of AD.

Mild cognitive impairment (MCI) is thought to represent the stage between normal forgetfulness due to aging and AD. Thus, MCI is a high risk factor for developing AD. However, due to heterogeneity of the MCI population the annual conversion rate varies from 4 to 31% between different studies/populations [2], [3], and thus predicting which MCI cases will actually convert to AD is still a challenge. According to a recent proposal about new research criteria for AD, the diagnosis of AD requires that the patient displays the core criterion of significant episodic memory impairment, and exhibits at least one or more of the supportive biomarker criteria [4]–[8]. Dozens of clinical measures and AD biomarkers have been proposed [9]. The diagnosis process involves collaborative efforts from neurologists, psychologists, radiologists, geneticists, and laboratories to interpret demographic information, neuropsychological tests, and biomarkers. Several studies have shown that by combining biomarkers one achieve an improvement in accuracy of the AD diagnosis [10], [11]. However, cognitive status does not always parallel the neuropathological changes due to the complex compensatory mechanisms present in AD. Therefore an accurate diagnosis of incipient/very early AD is not easy for the clinician, he/she is confronted by large amounts of quantitative and qualitative patient data, and particularly when much of the biomarker data may be ambiguous or even contradictory.

Recently, several computer-assisted support tools have been proposed as ways to help clinicians to make as accurate diagnoses as possible [12]–[14]. Decision support tools can provide objective and evidence-based information about the state of the patient; they are intended to integrate heterogeneous measurement data acquired from a patient in current clinical practice [12], [13]. There is evidence that computer-assisted analyses of patient data can achieve comparable diagnostic accuracy as experienced clinicians [12], [13]. The PredictAD tool can provide a classification and positions the patient into a continuous space between the values 0 and 1, indicating a patient’s disease state in relation to previously known control (healthy) and positive (disease) populations [12], [13]. This makes it possible to assess the disease severity i.e. it is not simply a yes/no diagnosis.

Many studies have been carried out to study the accuracy of biomarkers in detecting AD or predicting cognitive outcomes, however, there are few studies evaluating the relative importance of different biomarkers when they are used together. In some MCI cases, the biomarker data are ambiguous or contradict each another. It is unknown whether one of these biomarkers or their combination of them would be more sensitive, and whether quantitative values provide more information than a dichotomous rating [15]. In the present study, we grouped MCI cases from the Alzheimeŕs disease Neuroimaging Initiative (ADNI) cohort (http://adni.loni.ucla.edu/) into four groups: high likelihood, intermediate likelihood, uninformative likelihood, and low likelihood of converting to AD [5]. We evaluated the accuracies of predicting the AD diagnosis made by quantitative analysis using the computer assisted PredictAD tool [12] and by using current guidelines of prodromal AD [4]–[8] as identified by combinations of dichotomized cognitive scores and visual assessment of middle temporal lobe atrophy on MRI and dichotomized CSF biomarkers. Our working hypothesis was that computer-assisted analysis could help to improve accuracy of the diagnosis.

## Subjects and Methods

A total of 391 MCI cases were selected from the ADNI cohort (http://adni.loni.ucla.edu/). The demographics of the cases are summarized in Table 1. The definition of MCI is as follow: 1) subjects had Mini-Mental State Examination (MMSE) score between 24 and 30, 2) the memory complaint, 3) objective memory loss measured by education adjusted scores on Wechsler Memory Scale-Revised (WMS-R) Logical Memory II, 4) Clinical Dementia Rating (CDR) of 0.5, 5) the absence of significant levels of impairment in other cognitive domains, essentially preserved activities of daily living, and 6) the absence of dementia. All the cases had baseline ADNI cognitive testing results, including MMSE, Alzheimer’s Disease Assessment Scale-Cognitive subscale (ADAS-Cog), and several other common neuropsychological tests (http://adni.loni.ucla.edu/).

**Table 1:** Demographics and clinical examinations for the MCI patients.

	Non-AD converter (n = 233)	AD converter (n = 158)	p value
Gender Male/Female	158/75	95/63	0.044
Age years	75±8	74±7	0.544
Years of education	16±3	16±3	0.969
ApoE alle 4 carrier	66 of 198 (33%)	145 of 193 (75%)	<0.001
MMSE	27.3±1.8	26.7±1.7	0.001
RAVLT delayed recall	3.7±3.6	1.5±2.1	<0.001
RAVLT delayed recognition	10.3±3.5	8.7±3.6	<0.001
ADAS-Cog total score (11-item)	10.3±4.2	13.3±4.1	<0.001
ADAS-Cog total score (13-item)	16.7±6.1	21.6±5.4	<0.001
Clock drawing test	4.4±0.8	3.9±1.1	<0.001
Digit span forward	8.2±2.0	8.2±2.0	0.940
Digit span backward	6.2±2.2	6.0±1.8	0.523
Category fluency	16.3±4.9	15.3±4.8	0.048
Trail making test-A	41.8±20.1	49.7±25.9	0.001
Trail making test-B	115.7±67.5	151.1±67.5	<0.001
Digit symbol substitution test	38.5±11.2	33.8±11.0	<0.001
Scheltens scale	1.8±0.9 (n = 230)	2.2±0.9 (n = 157)	<0.001
Tau	93±61 (n = 115)	118±57 (n = 84)	0.004
Aβ1–42	178±58 (n = 115)	144±39 (n = 84)	<0.001

### Predicting AD Conversion with Current Prodromal AD Guidelines

The prediction of AD conversion was conducted with the combinations of clinical diagnosis of hippocampal pattern of memory loss [5] and biomarkers [16], [17]. The episodic memory loss of the hippocampal type, which is characterized by a free recall deficit on testing not normalized with cueing [5], was defined as present when the scores of delayed recall and delayed recognition of Auditory Verbal Learning Test (RAVLT) [18] were lower than 1 standard deviation of the corresponding mean values in healthy aged people, i.e. RAVLT delayed recall <3 and RAVLT delayed recognition <10 [19]. The Scheltens Scale was used to categorize the visual medial temporal lobe atrophy (MTA) on MRI, The scale rates atrophy on a 5-point scale (0 = absent, 1 = minimal, 2 = mild, 3 = moderate and 4 = severe) [16]. A single experienced neuroradiologist (YL) evaluated MTA in all of the cases. Scheltens score ≥3 was considered as having significant MTA. CSF levels of Tau >93 pg/ml, and Amyloid beta 1–42 (Aβ1–42) <192 pg/ml were considered as positive CSF markers [17]. The likelihood of AD conversion was defined as follows [5]:

- High likelihood: all clinical core criteria (RAVLT tests), Scheltens scale and CSF markers were positive,
- Intermediate likelihood: clinical core criteria was positive, one of MRI and CSF markers was positive, but the other one was lacking, i.e., not available,
- Uninformative likelihood: clinical core criteria was positive, and one of MRI and CSF markers was positive, but the other one was negative.
- Low likelihood: all clinical core criteria, Scheltens scale, and CSF markers were negative.

### Predict Conversion to AD with PredictAD Tool

The PredictAD tool [12] was used by one clinician who was blinded to the outcome during the evaluation. The PredictAD tool provided the rater with the available patient information at baseline, including demographics, apolipoprotein E (APOE) genotype, MMSE, ADAS-Cog, neuropsychological battery, MRI features automatically derived with FreeSurfer software package, and CSF laboratory analysis results. In addition, several features automatically derived from original MRI images using manifold learning [20], tensor-based morphometry [21], and hippocampus volume segmentation [22], developed in the PredictAD project (www.predictad.eu), were included. When determining with the assistance of PredictAD tool whether a subject had prodromal AD, the clinician based his opinion on presence of abnormal performances in the delayed recall and delayed recognition of Auditory RAVLT, the other neuropsychological tests were used as supportive evidences to determine the confidence of the clinical diagnosis. Given the baseline data, the clinician was then asked to categorize, i.e. diagnose, each patient into one of six categories: 1) clear indication of Non-AD, 2) probable indication of Non-AD, 3) subtle indication of Non-AD, 4) subtle indication of early AD, 5) probable indication of early AD, and 6) clear indication of early AD. One must emphasize that the clinician was asked to predict the diagnostic outcomes (Non-AD and AD converter) at the end of ADNI study using exclusively baseline data. To compare the accuracy of classification between automatically computed PredictAD diagnosis and clinician’s diagnosis with assistance of PredictAD tool, Disease State Index (DSI) values, computed by the PredictAD tool, were categorized uniformly between 0 and 1 as follows: (1) Clear indication of Non-AD: DSI <0.17, (2) Probable indication of Non-AD: 0.17≤ DSI <0.33, (3) Subtle indication of Non-AD: 0.33≤ DSI <0.50, (4) Subtle indication of early AD: 0.50≤ DSI<0.67, (5) Probable indication of early AD: 0.67≤ DSI <0.83, and (6) Clear indication of early AD: ≥0.83. In the automatically computed PredictAD diagnosis, all the neuropsychological and genetic tests, MRI, and CSF data were used to calculate the DSI.

To test the reproducibility of the diagnosis by clinicians with the assistance of PredictAD tool, interobserver variability and intraobserver reproducibility were analyzed. To test the interobserver variability, two clinicians (Y.L. and M.M.) independently made diagnosis in 40 (10%) randomly selected cases. To test the intraobserver reproducibility, one clinician made diagnosis in the 40 cases with an interval of at least 6 months between the diagnosis sessions.

### Statistical Analysis

The demographics and results of clinical exams were compared with Student t-test and chi square test between converters and non-converters. The conversion rates were calculated in cases with different likelihoods of AD conversion. The sensitivity, specificity, and accuracy of classification with the PredictAD tool, and different combinations of clinical scores, Scheltens scale, and CSF markers were calculated. McNemar’s test was used to compare the differences in accuracy produced with the PredictAD tool and the current AD guidelines. Kappa test was used to test interobserver variability and intraobserver reproducibility. The difference was considered statistically significant if p<0.05.

## Results

A total 387 of 391 MCI cases had undergone MRI exams, 199 MCI cases had undergone CSF examination, and 195 MCI cases had both MRI and CSF exams. During the 3-year follow-up, 158 of 391 (40%) converted to AD, 15 of 391 (4%) returned to normal cognitive status, and 218 MCI cases (56%) remained stable.

The conversion rates in different situations are summarized in Table 2.

**Table 2:** Conversion rates of baseline MCI in different situations.

		Criteria	Cases	Converters (percentage)
Baseline MCI			391	158 (40%)
Hippocampal pattern of memory loss (clinical)		Auditory Verbal Learning Test (RAVLT) +	136	72 (53%)
Core biomarkers				
	moderate to severe MTA	MRI +	92	51(55%)
	increased Tau or decreased Aβ1–42	Tau or Aβ1–42 +	150	76 (51%)
	increased Tau and decreased Aβ1–42	Tau and Aβ1–42 +	84	48 (57%)
High likelihood AD		RAVLT +, MRI +, CSF +	20	13 (65%)
Low likelihood AD		RAVLT − and biomarkers −	29	2 (7%)
Intermediate likelihood AD		RAVLT +, one biomarker +, and one not available	21	12 (57%)
	no Scheltens scale	RAVLT + and Tau or Aβ1–42 +	2	1 (50%)
	no CSF markers	RAVLT + and MRI +	19	11 (58%)
Uninformative likelihood AD		RAVLT +, one biomarker +, and one −	58	37 (64%)
	negative MRI	RAVLT + and Tau or Aβ1–42 +	41	24 (59%)
	negative CSF markers	RAVLT + and MRI +	17	13 (77%)

+ = positive finding.

Among the MCI cases who possessed a single positive marker (clinical core criteria or biomarker), those MCI cases who had increased Tau and decreased Aβ1–42 had the highest conversion rate (57%). The conversion rate for those MCI cases with Scheltens score≥3 was 55%. The MCI cases fulfilling the clinical core criteria for episodic memory loss evident both on free recall and recognition had the lowest conversion rate (53%).

As expected, the conversion rate was highest for those MCI subjects in high likelihood AD group (65%) and lowest for MCI subjects with low likelihood (7%). For the MCI cases with intermediate and uninformative likelihood of AD, the conversion rates were 57% and 64% respectively. Among the 20 baseline MCI cases estimated as high likelihood of AD, there were no significant differences in age, Scheltens score, concentrations of CSF Tau and Aβ1–42, AVLT scores, education years, gender, frequency of APOE e4 allele, or PredictAD DSI between converters (n = 13) and non-converters (n = 7) (p≥0.354).

### Sensitivity, Specificity, and Accuracy using Different Criteria and PredictAD Tool

The sensitivity, specificity, and accuracy of classification using the PredictAD tool and different criteria are listed in Table 3.

**Table 3:** Sensitivity, specificity, and accuracy (percentage) of classification between AD converters and non-converters with different combinations of examinations and use of the PredictAD tool (All MCI cases).

	Criteria	Sensitivity (95% CI)	Specificity (95% CI)	Accuracy
Neuropsychology tests (1)	Auditory Verbal Learning Test (RAVLT) +	46 (38–54)	73 (66–78)	62
Visual MTA (2)	MRI +	32 (25–40)	82 (76–87)	62
CSF (3a)	Tau or Aβ1–42 +	90 (82–96)	36 (27–45)	59
CSF (3b)	Tau and Aβ1–42 +	57 (46–68)	70 (60–78)	64
1+2		17 (12–24)	93 (89–96)	63
1+3a		44 (33–55)	78 (69–85)	64
1+2+3a		18 (11–28)	91 (84–96)	60
1+2+3b		4 (1–11)	97 (92–99)	58
PredictAD tool	Cutoff value of disease state index 0.50	73 (66–80)	71 (64–76)	72
Clinician with PredictAD tool assistance	Scale 1–3 stable MCI, scale 4–6 AD converter	75 (68–82)	68 (62–74)	71

The criteria of increased CSF Tau or decreased Aβ1–42 achieved the highest sensitivity (90%), but the lowest specificity (36%). The criteria that included episodic memory loss of the hippocampal type, Scheltens scale ≥3, increased CSF Tau, and decreases Aβ1–42 could correctly detect 111 of 115 non-AD converters, producing the highest specificity (98%), but the lowest sensitivity (6%).

The PredictAD tool produced the highest accuracy 72%, followed by the clinician’s diagnosis with the assistance of the PredictAD tool (71%). There was no significant difference in accuracy between the diagnosis by Predict tool alone and by the clinician (p = 1.0). The accuracy of the diagnosis by PredictAD tool alone was significantly higher than if one used the criteria of the biomarkers alone or combinations of clinical diagnosis of hippocampal pattern of memory loss and biomarkers (p≤0.037).

When considering the six categories of diagnosis (from clear indication of early AD to clear indication of non-AD), the interobserver variability and intraobserver reproducibility showed moderate agreements (kappa = 0.403, p<0.001; kappa = 0.462, p<0.001, respectively). However, when we simplified the six categories of diagnosis into AD and non-AD groups, excellent agreements were achieved (kappa = 0.800, p<0.001 for interobserver variability; kappa = 0.850, p<0.001 for intraobserver reproducibility).

The PredictAD DSI achieved accuracy of 81% in detecting non-AD converters, and an accuracy of 63% in detecting AD converters. In the clinician’s diagnosis with the assistance of the PredictAD tool, the accuracies were 80% and 62% respectively. However, with the assistance of PredictAD tool, the clinician’s diagnosis of high confidence (clear non-AD, probable non-AD, probable AD, and clear AD) was dramatically improved compared to the PredictAD tool alone. The number of non-AD diagnoses made by the clinician with high confidence increased from 118 to 146 (from 30% to 37%), and the number of AD diagnosis with high confidence increased from 87 to 112 (from 22% to 29%). With help of the PredictAD tool, the clinician made diagnoses of clear non-AD or clear AD in 144 of 391 (37%) cases with overall accuracy of 84% (Tables 4, 5, 6).

**Table 4:** Sensitivity, specificity, and accuracy (percentage) of classification between AD converters and non-converters with different combinations of examinations and use of the PredictAD tool (195 MCI cases with both MRI and CSF results).

	Criteria	Sensitivity (95% CI)	Specificity (95% CI)	Accuracy
Neuropsychology tests (1)	Auditory Verbal Learning Test (RAVLT) +	48 (37–59)	70 (60–78)	61
Visual MTA (2)	MRI +	31 (22–43)	83 (75–89)	61
CSF (3a)	Tau or Aβ1–42 +	90 (81–95)	36 (27–45)	59
CSF (3b)	Tau and Aβ1–42 +	57 (45–67)	71 (61–79)	65
1+2		19 (12–30)	94 (87–97)	62
1+3a		43 (33–55)	79 (70–86)	64
1+2+3a		18 (11–28)	91 (84–95)	60
1+2+3b		4 (1–11)	97 (92–99)	57
PredictAD tool	Cutoff value of disease state index 0.50	76 (65–84)	71 (61–79)	73
Clinician with PredictAD tool assistance	Scale 1–3 stable MCI, scale 4–6 AD converter	78 (68–86)	68 (58–76)	72

**Table 5:** Accuracy of classification between AD converters and non-converters with the PredictAD tool.

	Final Diagnosis			Total	Accuracy
	AD	Healthy	MCI		
Clear indicationof non-AD	2	9	43	54 (14%)	96%
Probable indicationof non-AD	9	4	51	64 (16%)	86%
Subtle indicationof non-AD	27	2	53	82 (21%)	67%
Indication of Non AD	38	15	147	200 (51%)	81%
Subtle indicationof AD	58	0	46	104 (27%)	56%
Probable indicationof AD	51	0	20	71 (18%)	72%
Clear indication of AD	11	0	5	16 (4%)	80%
Indication of AD	121	0	70	191 (49%)	63%

Note: Clear non-AD: disease state index <0.17, Probable non-AD: 0.17≤ disease state index <0.33, Subtle non-AD: 0.33≤ disease state index <0.50, Subtle AD: 0.50≤ disease state index <0.67, Probable AD: 0.67≤ disease state index <0.83, Clear AD: disease state index ≥0.83. ‘Healthy’ denotes MCI cases which converted back to the category ‘healthy’ during the study and belong still to the non-AD group. Overall accuracy of diagnosis was 72%.

**Table 6:** Accuracy of classification between AD converters and non-converters the clinician making the diagnosis with assistance of the PredictAD tool.

	Final Diagnosis			Total	Accuracy
	AD	Healthy	MCI		
Clear indicationof non-AD	6	12	64	82 (21%)	93%
Probable indicationof non-AD	15	3	46	64 (16%)	77%
Subtle indicationof non-AD	18	0	34	52 (13%)	65%
Indication of Non AD	39	15	144	198 (50%)	80%
Subtle indicationof AD	43	0	38	81 (21%)	53%
Probable indicationof AD	31	0	19	50 (13%)	62%
Clear indication of AD	45	0	17	62 (16%)	73%
Indication of AD	119	0	74	193 (50%)	62%

Overall accuracy of diagnosis was 71%.

The clear AD diagnoses (16 cases) in the PredictAD DSI index included 5 stable MCI cases. The Probable indication of AD (71 cases) in the PredictAD DSI index included 20 stable MCI individuals. Among this subgroup there were no significant differences in age, gender, presence of APOE 4, years of education, concentrations of CSF markers, Scheltens scores, MMSE, or RAVLT results between AD converters and those with stable MCI (p≥0.236).

Because a variety of subject-specific factors may be influencing results in unkown ways, we also performed analyses on a subset of 195 participants who had all data available (neuropsychology, MRI and CSF) and repeated the analyses reported in Table 4 for this subset. The sensitivities, specificities, and accuracies of classifications using the PredictAD tool and different criteria in this subgroup were highly similar to those in whole group (Tables 3–4).

## Discussion

The results show that the PredictAD tool alone (72%) and the clinician with the assistance of the PredictAD tool produced comparable or higher accuracy in predicting 3-year MCI outcome than current research criteria for diagnosis of prodromal AD. The literature is somewhat confusing, due to differences in size of study populations, statistical methods, and length of follow-up etc., but it seems that the overall accuracy of combinations of clinical data and/or biomarkers in predicting AD conversion from MCI has varied from 67% to 93% [23]–[28]. Liu et al., using the 100 MCI cases from AddNeuroMed data and a combination of neuropsychological tests and structural MRI biomarkers reported overall accuracy 69% during one year follow-up [27]. Studies with the ADNI cohort reported accuracies 67–77% when using combinations of clinical measures and CSF and MRI biomarkers [23], [24], [26].

We acknowledge that the prediction accuracy of about 70% is not high concerning the clinical utility but the result is still comparable with the current state-of-the-art. It reflects a reality that the current prodromal AD guidelines and combinations of biomarkers are not perfect. However, our point was not to develop a novel method but to show how the current guidelines compare with computer-assisted methods. The PredictAD tool can provide objective and evidence-based information about the state of the patient by integrating heterogeneous measurement data acquired from a patient in current clinical practice. PredictAD makes it possible to assess the disease severity, i.e. it is not simply a yes/no diagnosis. Itś graphical user interface can make it easy for clinician to explore every single test or biomarker, giving more confident to clinicians than a probability or yes/no diagnosis calculated with certain software with underlying complex statistical calculation. Using the PredictAD tool, the clinician was able to detect a sub-population for which the accuracy was 84% which starts to be high enough for affecting the clinical reasoning. It is good to remember that 100% is not the correct target value in reality due to different reasons: 1) Stable MCI and progressive MCI cases in ADNI are not pathologically confirmed cases. It has been shown in different studies that the agreement of the clinical and neuropathology diagnoses is 70–90% [29]–[31]. In other words, even 72% is within this range and studies reporting values >90% should be interpreted with a caution. 2) Even neuropathological diagnoses are not perfect.

**Figure 1:**
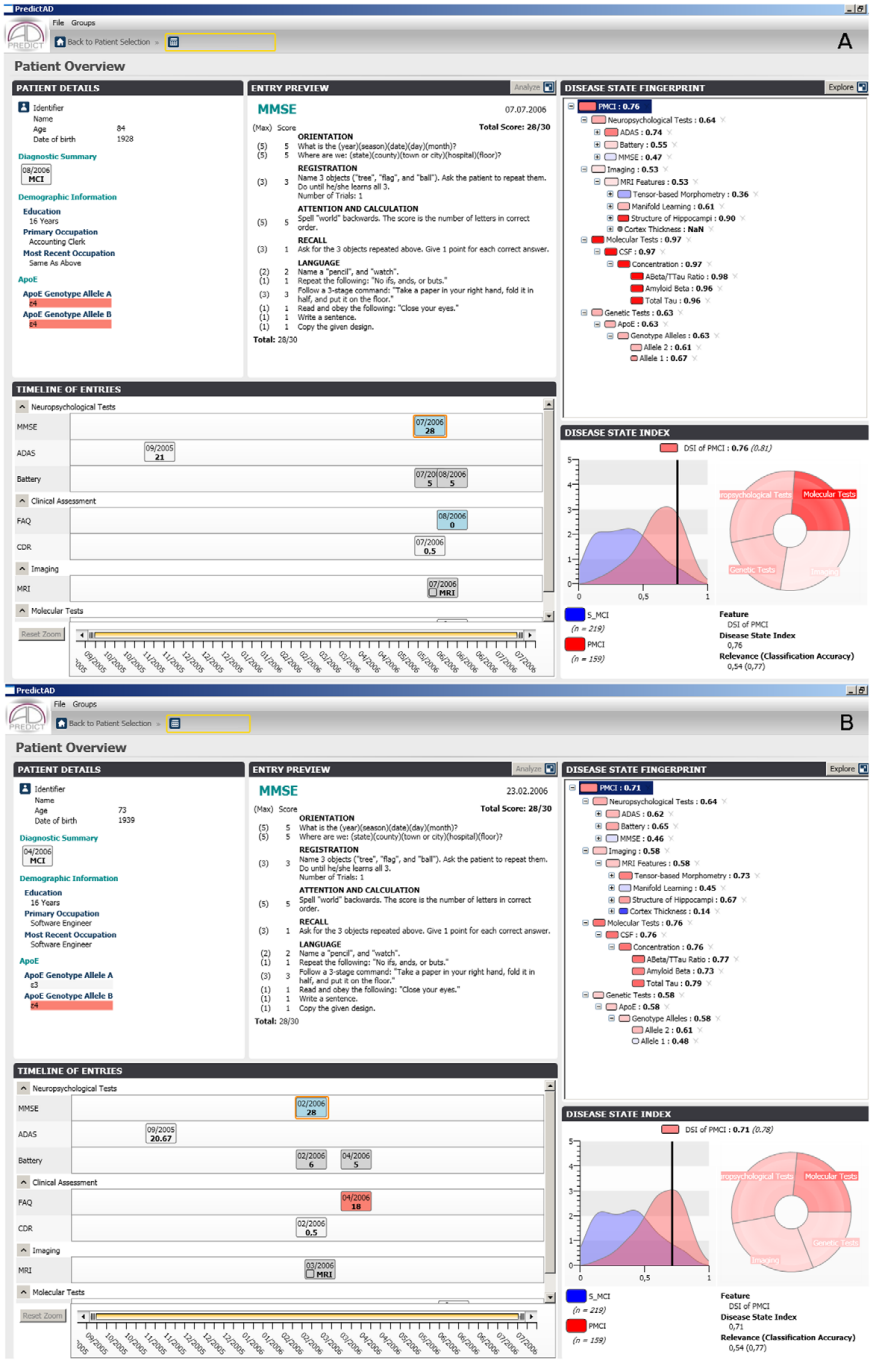
Screenshots from the PredictAD tool for two cases. The cases A and B had similar baseline neuropsychological tests, biomakers, and genetic tests, but the case A did not convert to AD, case B converted to AD during 3-year follow-up period. The case A was classified by both predictAD tool and current guildline for prodromal AD. It is probable that this case will convert in longer follow-up. The MCI subjects like case A seem to be a potential interesting study group. It might be possible to identify sensitive biomarkers to detect AD at early phase or explore novel preventative factors to delay the onset of symptoms of AD by investigating this subgroup. The main window of the PredictAD tool consists of five panels. The ‘Patient details’ panel shows basic information about the patient. The ‘Timeline of entries’ panel contains information about all measurements acquired from patient. The panel is interactive: the user can click any of the entries visible and a summary isshown in the ‘Entry preview’ panel. The disease state fingerprint is shown in the ‘Disease state fingerprint’ panel. When the user selects any of the item from the fingerprint, details behind the item are shown in the ‘Disease state index’ panel. The distributions show the probability density functions of the corresponding item for the study and control groups, in this case PMCI and SMCI groups, and the value measured from the patient is shown by a vertical black line.

It is interesting that about 30% MCI cases with clear (DSI ≥0.83, 5 of 16 cases) and probable (0.67≤ DSI <0.83, 20 of 71 cases) indications of AD did not convert to AD during the 3-year follow-up, even though they did not significantly differ from AD converters in age, gender, presence of APOE4, years of education, concentrations of CSF markers, Scheltens scores, MMSE, and RAVLT results (Figure 1). The reason why those 25 stable MCI cases did not convert to AD is still unknown. In fact, this subgroup population seems to be interesting, and a detailed investigation of this subgroup, we might uncover novel preventative factors which delay the onset of symptoms of AD.

Current research criteria for prodromal AD [4] emphasizes that the core criteria of episodic memory impairment should not only include deficit on delayed free recall but also on cued recall or recognition. In this paper we used RAVLT free recall and recognition scores to form the criteria of episodic memory impairment. Adjustments for gender or education were not used, and in addition it can be argued that results may have been different if another memory test or cut-off values would have been used. However, it is essential to remind that all MCI subjects in ADNI cohort already fulfilled a significant memory impairment measured with WMS-R logical memory II test (with education correction). Thus subjects who fulfilled the criteria of episodic memory impairment in the present paper performed lower than expected for age altogether in three memory tests.

It has been shown that the Scheltens scale can classify AD patients and healthy controls or other types of dementia with high sensitivity, specificity, and accuracy [16], [32], [33]. Westman et al. [34] applied Scheltens scale 2 and 3 as cutoff values in 101 MCI cases from the multicenter study AddNeuroMed study. They reported that the visually evaluated atrophy of MTL produced similar accuracy in predicting conversion from MCI to AD (68%) compared to multivariate regional MRI classification and manual hippocampal volumes at one year follow-up. We applied Scheltens scale 3 as the cutoff value in the ADNI data and found prediction accuracy (62%) during the 3-year follow-up.

In the present study, according to the most recent criteria for likelihood of AD, only 25 cases fulfilled the high likelihood of AD, i.e. all clinical core criteria, MRI and CSF markers were positive, fifteen of those 25 (60%) cases did convert to AD. Moreover, very low sensitivities (6%–57%) were achieved by using the combination of clinical core tests, and MRI and CSF markers. In contrast, by using the PredictAD tool, the number of clinician’s diagnosis of a clear indication of AD was 62 cases, and 45 of those 62 (73%) cases did convert to AD. This finding indicates that the PredictAD tool uses the clinical, MRI, and CSF data in a much more efficient way than the recent criteria applied with specific cut-off values for making the diagnosis of AD.

We acknowledge that the present study has certain limitation. In the predicting AD conversion with current prodromal AD guidelines, only RAVLT tests were used to define if the subjects had prodromal AD symptoms, but in the predicting AD conversion with PredictAD tool alone, all the neuropsychological tests were used. When the clinician determined if the subjects had prodromal AD symptoms with the assistance of PredictAD tool, only RAVLT tests were used as in the predicting AD conversion with prodromal AD guidelines. However, the clinician was not blinded to the other neuropsychological tests, the performance at the other tests exploring cognitive domains other than memory were used to increase the confidence of clinical diagnosis. The overall predicting accuracy was 72%, 71%, and 64% for the PredictAD tool alone, clinician’s prediction with the assistance of the PredictAD tool, and the best combination of the core clinical and biomarkers respectively. Diagnosis with the PredictAD tool was significantly more accurate than diagnosis by biomarkers alone or the combinations of clinical core criteria and biomarkers. The methods judging if a subject presented prodromal symptoms were not equal. It may explain the differences in overall predicting accuracy. The findings imply that a single neuropsychological test is not powerful enough to replace the other neuropsychological tests in early AD diagnosis, enhancing the justification of using PredictAD tool in clinical practice.

In conclusion, with the assistance of the PredictAD tool, the clinician can predict AD conversion more accurately than than the current research criteria for prodromal AD.
